# Supplementary material for: Anomalous normal fluid response in a chiral superconductor UTe2
Source: Nat Commun. 2021 May 11;12:2644. doi: 10.1038/s41467-021-22906-6 (PMC8113495; doi:10.1038/s41467-021-22906-6)
Supplement: Supplementary file 1 — Supplementary Information [file 41467_2021_22906_MOESM1_ESM.pdf]

Supplementary Information : Anomalous normal fluid response in a chiral superconductor UTe_2

CONTENTS

Supplementary Note 1: Microwave surface impedance measurement procedure 2
A. Transmission measurement procedure 2
B. Determination of resonance properties f_0 and Q 4
C. Converting resonance properties (f_0 and Q) into surface impedance (Z_s) 5

Supplementary Note 2: Types of microwave resonators 6
D. Disk dielectric resonator 6
E. Hollow cylinder dielectric resonator (HDR) with hot finger 9

Supplementary Note 3: Normal state $Z_s(T)$ and scattering lifetime 10

Supplementary Note 4: Estimation for the mean free path and justification for local electrodynamics 13

Supplementary Note 5: Inconsistency between singlet pairing states and low temperature behavior of the penetration depth 14

Supplementary Note 6: Further qualitative evidence of the residual normal fluid through the two-fluid model 15

Supplementary Note 7: Remarks on the residual specific heat result 17

Supplementary Note 8: Details of the fitting parameter in the normalized superfluid density analysis 18

Supplementary Note 9: Insulating properties of Uranium oxides 20

Supplementary Note 10: Effect of N-grease on the surface impedance study of the sample 20

Supplementary Note 11: Examination of topologically trivial origins of the surface normal fluid 21

References 23

SUPPLEMENTARY NOTE 1: MICROWAVE SURFACE IMPEDANCE MEASUREMENT PROCEDURE

A. Transmission measurement procedure

The surface impedance Z_s of a superconducting sample can be obtained from both resonant techniques and non-resonant techniques. In this work, Z_s of the UTe_2 sample was measured by a resonant technique. Readers who are interested in the non-resonant techniques can refer to Ref. [1, 2]. For the resonant techniques, one can obtain Z_s from the resonance properties (resonant frequency f_0 and quality factor Q) through either a microwave transmission or reflection measurement that involves the sample of interest. Here, we conducted a transmission measurement to obtain Z_s of the sample.

For a microwave transmission measurement, the input and output microwave signals are generated and received by the Keysight vector network analyzer (VNA) N5242A (Supplementary Fig. 1). The VNA calculates a voltage ratio of the signal at its input (port 1) and output port (port 2) to obtain the complex microwave transmission scattering parameter S_{21}^{tot} in the frequency domain. The generated signal from port 1 goes into the microwave transmission line (blue lines in Supplementary Fig. 1) of the BlueFors XLD dilution fridge and reaches down to the resonator attached to the mixing chamber plate (MXC) whose base temperature is 11 mK. The signal passed through the resonator comes back to port 2 of the VNA. The details of the two resonator designs used in this study will be discussed in Supplementary Note 2.

The S_{21}^{res} measured by the VNA includes cable loss and phase shift from the transmission lines. For high-Q resonant techniques, the phase variation with frequency from the cable near the resonant frequency is typically small (< 0.01 radian for the 3dB bandwidth of the resonance in our experimental setup in Supplementary Note 2.D.), but the cable loss (decrease in the magnitude of S_{21}) can contribute a large portion to $|S_{21}^{\mathrm{tot}}|$. One needs to subtract out the cable loss in order to obtain the magnitude of the scattering parameter

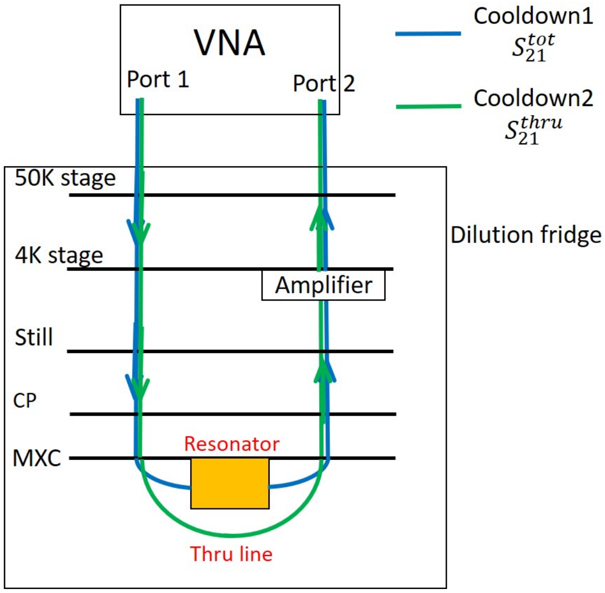

Supplementary Fig. 1. (Blue) schematic view of the signal path of the microwave transmission measurement S_{21}^{tot} that includes the resonator. (Green) the signal path of the thru line transmission measurement S_{21}^{thru} which shares the same signal path except that it bypasses the resonator, and is measured in a separate cooldown. The thru line measurement S_{21}^{thru} gives a measure of the cable loss.

purely from the resonator. For this correction, an additional transmission line measurement called a thru line measurement is used. The thru line measurement follows the same signal path utilizing the same coaxial cables but bypasses the resonator, and is measured in a separate cooldown. The measured transmission from this correction line is called thru-S_{21} (S_{21}^{thru}). By subtracting $|S_{21}^{\mathrm{thru}}|$ from $|S_{21}^{\mathrm{tot}}|$, one can estimate the magnitude of the resonator transmission $|S_{21}^{\mathrm{res}}|$,

$$|S_{21}^{\mathrm{res}}| = |S_{21}^{\mathrm{tot}}| - |S_{21}^{\mathrm{thru}}|. \tag{1}$$

With the above procedure for finding S_{21}^{res}, broadband transmission (6 $\sim$ 20 GHz, the range is limited by the microwave amplifier) is first measured to locate a resonant peak of interest. For the located peak, a narrow band measurement is done to precisely resolve the sharp $S_{21}^{\mathrm{res}}(f)$ near the peak of the resonance.

B. Determination of resonance properties f_0 and Q

With the measured resonator $S_{21}^{\text{res}}(f)$ curve, one can characterize the resonance properties. There is the resonant frequency f_0, which is related to the reactance of the resonator ($f_0 \sim 1/\sqrt{LC}$ where L is the inductance and C is the capacitance of a lumped-element equivalent circuit representation of the resonator), and the quality factor Q which is related to the microwave dissipation P_{diss} in the resonator ($1/Q \sim P_{\text{diss}}$). The extraction of f_0 and Q can be done by fitting $S_{21}^{\text{res}}(f)$ curve near the resonance by a "Phase versus frequency fit" [3] which models the phase ϕ of the complex $S_{21}^{\text{res}}(f) = |S_{21}^{\text{res}}(f)|e^{i\phi(f)}$ with,

$$\phi(f) = \phi_0 + 2\tan^{-1}\left[2Q_L\left(1 - \frac{f}{f_0}\right)\right], \tag{2}$$

where Q_L is a "loaded" quality factor and ϕ_0 is a phase offset. The error bars of the f_0 and Q are determined by a deviation of f_0 and Q from the estimated values, which increases the root-mean-square error σ_{RMS} of the fit by 1 %. As described in Ref. [4], the main source of the error bar of f_0 and Q is the noise in $S_{21}(f)$ data. If the signal-to-noise ratio of S_{21} is large (small), which makes the $S_{21}(f)$ curve well (poorly) defined, then f_0 and Q can have a narrower (wider) range of values while giving fits with similar values of σ_{RMS}. The error bars of the f_0 and Q are propagated into that of the Z_s using the relation Eq. (10-12).

Ideally, the inverse of the quality factor $1/Q$ should describe the dissipation from the resonator itself. However, to excite and pick up the microwave signal, the resonator should be coupled to the external world through excitation/pick-up loops. This coupling causes some portion of the energy in the resonator to leak out to the external world [5], adding an additional source of energy dissipation which is described by $1/Q_c$ where Q_c is called the coupling Q. The Q arising purely from losses within the resonator is called the unloaded Q (Q_u). The Q factor obtained from fitting the raw S_{21} data above is called the loaded Q (Q_L) because it describes the dissipation from both the resonator itself $1/Q_u$ and the coupling $1/Q_c$,

$$1/Q_L = 1/Q_u + 1/Q_c. \tag{3}$$

To obtain information on the microwave dissipation purely from the resonator, one needs to know Q_u which removes the effect of coupling. In the case of equal coupling from the input and output side, Q_u can be calculated as [6, 7],

$$Q_u = \frac{Q_L}{1 - |S_{21}(f_0)|}. \tag{4}$$

To obtain the dissipated microwave power from the sample P^s_{diss}, which gives the resistance R_s of the sample, one needs to further subtract the dissipated power from the background components P^b_{diss} of the resonator. Note that the typical sources of the background dissipation of the resonator are Ohmic dissipation from the enclosure of the resonator (if it is normal metal), dielectric loss from the dielectric materials, and radiation loss from any openings of the resonator. The resonator dissipation and the unloaded Q can be written as,

$$P^{\text{tot}}_{\text{diss}} = P^s_{\text{diss}} + P^b_{\text{diss}}, \tag{5}$$

$$\frac{1}{Q_u} = \frac{1}{Q_s} + \frac{1}{Q_b}. \tag{6}$$

To remove the background contribution on the dissipation and Q, two consecutive measurements are required. One is the measurement without the sample ($Q_u^{\text{w.o.}}$) which measures the background dissipation. The second one is the measurement with the sample inserted in the resonator ($Q_u^{\text{w.}}$) which now includes the actual sample properties. The sample quality factor $Q_s(T)$ can be finally determined by the results of those two measurements [8],

$$1/Q_u^{\text{w.o.}} = 1/Q_b, \tag{7}$$

$$1/Q_u^{\text{w.}} = 1/Q_b + 1/Q_s, \tag{8}$$

$$1/Q_s(T) = 1/Q_u^{\text{w.}}(T) - 1/Q_u^{\text{w.o.}}(T). \tag{9}$$

Once the subtraction is done, it leaves only the sample contribution to the quality factor Q_s.

C. Converting resonance properties (f_0 and Q) into surface impedance (Z_s)

Once $f_0(T)$ and $Q_s(T)$ are obtained, the surface impedance $Z_s = R_s + iX_s$ of the sample can be determined as [9],

$$R_s(T) = G_{\text{geo}}/Q_s(T) \tag{10}$$

$$\Delta X_s(T) = -2G_{\text{geo}}\frac{\Delta f_0(T)}{f_0(T_{\text{min}})}. \tag{11}$$

$$G_{\text{geo}} = \frac{\omega\mu_0 \int_V dV|H(x,y,z)|^2}{\int_S dS|H(x,y)|^2}. \tag{12}$$

Here, $\Delta f_0(T) = f_0(T) - f_0(T_{\text{min}})$, $\Delta X_s(T) = X_s(T) - X_s(T_{\text{min}})$ where T_{min} is the minimum temperature of the measurement, $\int_V dV$ represents the integration over the volume of the

resonator, and $\int_S dS$ represents the integration over the surface of the sample. $G_{\rm geo}$ is the sample geometric factor which can be calculated either analytically using the field solution inside the resonator for each resonant mode (if the solution is available), or experimentally by comparing the experimentally measured R_s from Supplementary Equation (10) with the calculated $R_s = \sqrt{\mu\omega\rho_{\rm dc}/2}$ from electrical resistivity $\rho_{\rm dc}$ of a dc transport measurement in the normal state. Note that this equality is valid in the large scattering rate limit ($\omega\tau \ll 1$. Here, $\omega = 2\pi f$ and τ is the scattering lifetime of the electron) in the normal state of the sample [10]. This limit can be reached by increasing the sample temperature well above T_c which typically reduces the scattering lifetime τ. Once the limit is reached, $G_{\rm geo}$ can be determined.

Also, the absolute value of $X_s(T)$ can be determined in the following way. When the $\omega\tau \ll 1$ regime is reached in the normal state, the complex conductivity $\tilde{\sigma} = ne^2\tau/m^*(1 + i\omega\tau) \approx ne^2\tau/m^*$ only has a real part. As $Z_s = \sqrt{i\mu\omega/\tilde{\sigma}}$, purely real $\tilde{\sigma}$ results in $R_s = X_s = \Delta X_s(T) + X_{s0}$. From the comparison of R_s and ΔX_s, the offset in the sample reactance X_{s0} can be found, which yields the absolute value of $X_s(T)$ for the full temperature range. With the absolute values of $R_s(T)$ and $X_s(T)$ determined, one can calculate the microwave complex conductivity $\tilde{\sigma}(T)$ whose real and imaginary parts provide useful information about the normal fluid and superfluid response of a superconductor.

One important question here is, what is the unambiguous experimental signature that one has achieved the $\omega\tau \ll 1$ regime in the normal state? Suppose the $\omega\tau \ll 1$ regime is achieved above a certain temperature $T_{ls} > T_c$. For $T > T_{\rm ls}$, $R_s(T) = X_s(T)$ so their temperature derivative should also satisfy $dR_s/dT = dX_s/dT$ [11]. This means that once a temperature above which $dR_s/dT = d(\Delta X_s)/dT$ is satisfied (note that $dX_s/dT = d(\Delta X_s)/dT$), $T_{\rm ls}$ can be identified. Note that the relative magnitude of the temperature derivative of R_s and X_s has $G_{\rm geo}$ as a common factor, thus the value of $G_{\rm geo}$ does not affect this comparison test.

SUPPLEMENTARY NOTE 2: TYPES OF MICROWAVE RESONATORS

D. Disk dielectric resonator

To measure the surface impedance Z_s and the corresponding microwave complex conductivity $\tilde{\sigma}$ of the UTe_2 single crystal sample with the procedures described in Supplementary

Note 1, two types of resonator design were used. The first type is the disk dielectric resonator (Supplementary Fig. 2). The disk dielectric resonator (disk DR) consists of a cylindrical rutile disk with a diameter of 3 mm, and height of 2 mm. With a high in-plane dielectric constant over 110 below 20 K [12], the rutile disk facilitates its fundamental resonant mode (TE_{011}) at $f_0 \approx 11$ GHz [13]. In this mode, the disk induces a radial microwave magnetic field on the sample surface, which induces an azimuthal circulating current on the sample surface. Here, the UTe_2 sample is mounted with an ab-plane facet in contact with the top of the rutile disk. Therefore, the measured Z_s data in the main text represents ab-plane electrodynamics. Note that to avoid the effect of microwave heating, we measured f_0 as a function of input microwave power P_{in}, and found that f_0 is independent of P_{in} below -30 dBm (measured at the source). We used $P_{\text{in}} = -40$ dBm to produce the $Z_s(T)$ data. In this case, the injected microwave power at the input coupling loop of the resonator was -51 dBm. Electromagnetic simulation (HFSS) of the resonator and sample provides an estimation of the microwave magnetic field intensity at the sample surface of $\approx 1\mu$T.

To cut down the background dissipation and obtain a resonant microwave transmission with a higher qualify factor, the top and the bottom side of the disk is enclosed by a niobium plate and film. This niobium top and bottom plate structure significantly reduces the background Ohmic loss because they are superconducting, and they have temperature-independent properties in the superconducting temperature range for UTe_2. Also the semi-cavity niobium structure reduces the radiation loss to a marginal level. The cutoff frequency of wave propagation through the $\approx$ 3 mm diameter aperture and the side space is 58.6 GHz. These cutoffs are much higher than the measurement frequency of 11 GHz, and hence the radiation field cannot propagate. Lastly, the dielectric loss should be marginal too since the loss tangent $\tan\delta = 1/Q_{\text{diel}}$ is $\sim 10^{-8}$ for the rutile below 10 K [12]. With the design to reduce the background loss mechanism, the high background quality factor Q_b (low background loss) was obtained $Q_b \approx 10^5$. This Q_b was subtracted from the measured Q_u with the sample present (Supplementary Fig. 2(b)), as described in Supplementary Equation 9.

Once the background measurement was done, the sample was inserted and thermally glued by N-grease. The temperature dependent resonance properties $\Delta f_0(T) = f_0(T) - f_0(50\text{mK})$ and $Q(T)$ which involves the sample were measured for the temperature range of 50 mK to 3 K. The temperature range is limited because above 3 K, the niobium semi-cavity

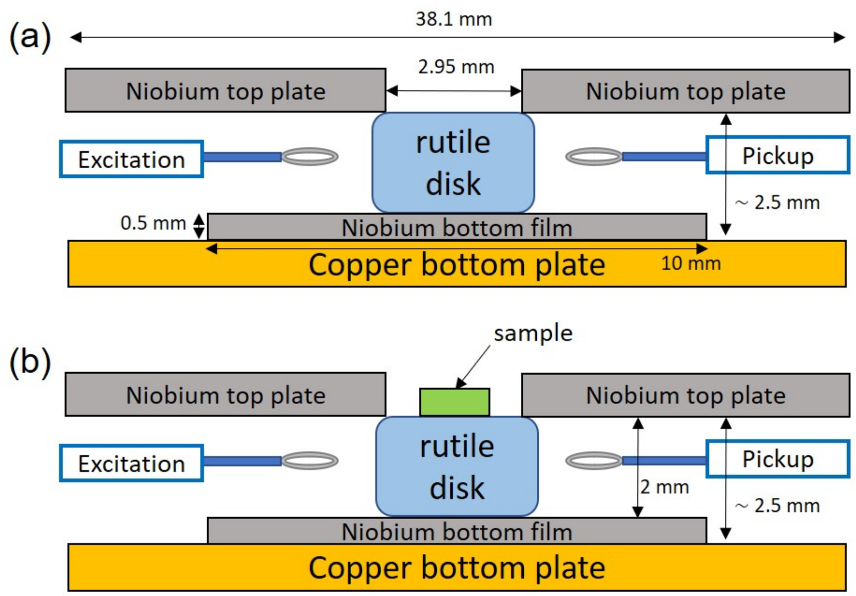

Supplementary Fig. 2. Cross-section view of the cylindrically-symmetric disk dielectric resonator setup to facilitate TE_{011} resonance mode at $\approx$ 11 GHz (a) without the sample for the background measurement (b) with the sample for the sample Z_s measurement.

structure starts to show finite temperature dependence on its contribution to Δf_0 and Q.

Once the $\Delta f_0(T)$ and $Q_s(T)$ were determined, they were converted to $R_s(T)$ and $\Delta X_s(T)$ with temporary value of $G_{geo} = 1$. To determine the absolue value of the $X_s(T)$, one needs to find the temperature range of the $\omega\tau \ll 1$ regime in the normal state. To do so, $R_s(T)$ and $\Delta X_s(T)$ curves were compared and overlaid. As one can see from Fig. 1 of the main text, both curves show the very same slope in the 2 to 3 K range. This suggests that the $\omega\tau \ll 1$ regime is reached, and the absolute value of $X_s(T)$ was determined by $R_s(T) = X_s(T)$ between 2 and 3 K. Then, the R_s at 3 K, which involves the undetermined G_{geo}, was compared to the R_s calculated from the electrical resistivity obtained from the dc transport measurement [14]. Note that there is 1:2 anisotropy in the value of the resistivity along the a and b-axis in the above temperature range. The two resistivity values were averaged with the weighting factor ratio 3:7, which is an inverse of the ratio of the dimensions of the sample in each direction. In this way, G_{geo} was determined to be 713 Ω.

However, one may be concerned that the 2 to 3 K interval is not wide enough to correctly identify the convergence between the slope of the $R_s(T)$ and $X_s(T)$ curves. For a more rigorous check, we adopted the hollow cylinder dielectric resonator design similar to Ref. [8]

with the hot finger technique [15–17] to measure $R_s(T)$ and $X_s(T)$ up to 20 K, where the $\omega\tau \ll 1$ regime is safely reached in the cases of several other heavy fermion superconductors [18, 19].

E. Hollow cylinder dielectric resonator (HDR) with hot finger

The key for measuring $Z_s(T)$ of the sample in the normal state up to a high temperature where τ becomes small enough (i.e. 20 K) is to fix the background contribution to the f_0 and Q while the sample temperature is varied. To accomplish this goal, two main features are introduced in the HDR. First, the niobium structure, which contributes a strong temperature dependence to f_0 and Q above 3 K, was replaced with copper walls (Supplementary Fig. 3). Copper shows only a small change (< 3 %) in its electrical resistivity between 4 and 20 K [20], cutting down the background temperature dependence on Q to a manageable level. The copper walls were moved away from the sample and the rutile to minimize the contribution from the walls to the quality factor.

Secondly, the hot finger technique [15–17] was introduced to decouple the sample stage temperature and the resonator background temperature (rutile, copper walls). This temperature decoupling was designed especially because the rutile cylinder has a finite temperature dependence in its dielectric constant above 10 K [12], which can give a background temperature dependence in f_0. The hot finger structure utilizes Vespel SP-1 rods to thermally isolate the resonator (which is directly thermally anchored to the cold plate at $\sim$ 100 mK) and the thermal reservoir (anchored to the mixing chamber of the fridge with thermal straps). The thermal reservoir and the sample thermal stage are weakly connected by a non-magnetic stainless steel tube (304 SS). This "weak-link" provides a certain degree of thermal decoupling during the sample temperature ramping, while the cooldown time is still in a reasonable range. A 1 mm diameter single crystal sapphire rod is then attached to the sample thermal stage. At the other end of the sapphire rod, the sample is mounted with GE varnish, and placed in the middle of the hollow rutile cylinder where the microwave magnetic field intensity is the highest. The 3 mm diameter cylindrical bore of the 10 mm outer diameter and 10 mm height hollow rutile cylinder ensures no mechanical, and hence no thermal contact between the sample and cylinder. The thermometer is attached to the sample thermal stage and the resistive heater is wound around the sapphire rod to increase the sample

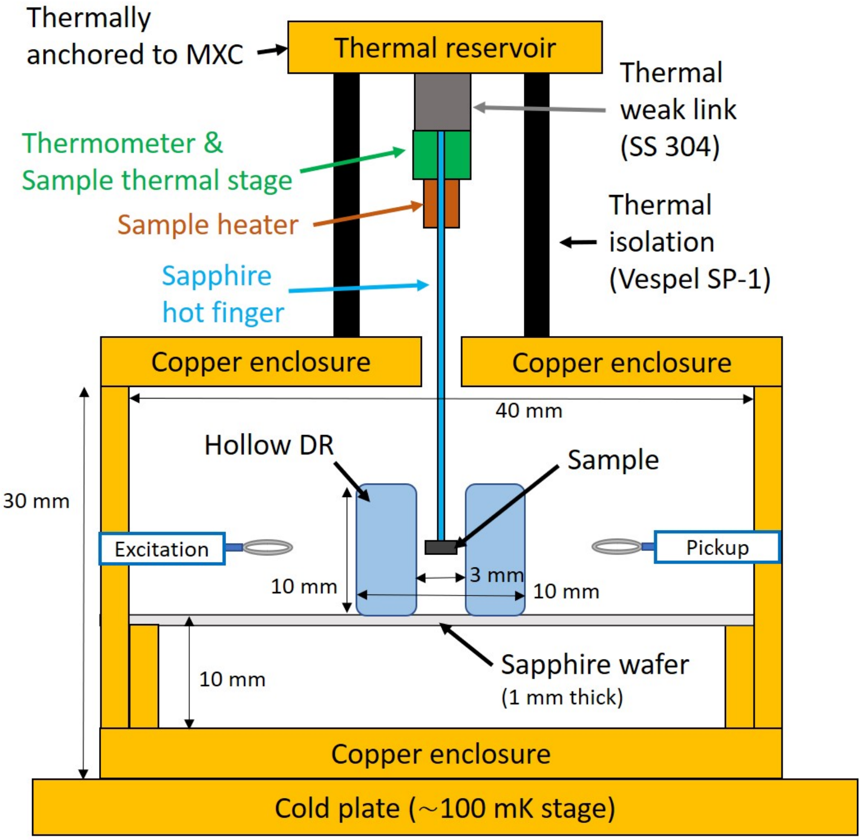

Supplementary Fig. 3. Cross-sectional view of hollow rutile cylinder dielectric resonator (HDR) and the attached sapphire hot finger setup. The base temperature of the sample thermal stage is ∼220 mK.

temperature. The heater wires are twisted to cancel the dc magnetic field from the current through them. With this hot finger design, when the sample temperature is increased to 20 K, the resonator temperature only increases to 620 mK. With the two changes made in the HDR design, it successfully decouples and minimizes the background contribution to the resonance properties. The data taken from the HDR is discussed in Supplementary Note 3.

SUPPLEMENTARY NOTE 3: NORMAL STATE $Z_s(T)$ AND SCATTERING LIFETIME

With the UTe_2 sample (the same one used in the disk DR) mounted in the HDR, the Z_s data of the sample was obtained from 220 mK to 20 K. The resonant mode under study (TE_{025}) has a resonant frequency f_0 at ≈ 9.07 GHz, which is the closest to f_0 of the resonant

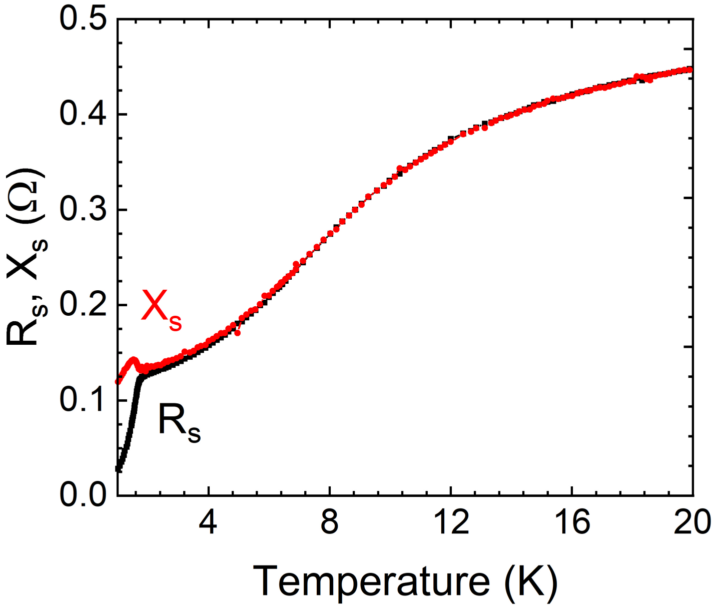

Supplementary Fig. 4. Measured $Z_s(T)$ data at 9.07 GHz of the UTe_2 sample obtained from the HDR.

mode of the disk DR (11 GHz). The obtained Z_s is displayed in Supplementary Fig. 4. As one can see, the slope of the R_s and X_s curves are already quite similar above T_c, and completely merge above 5 K conservatively speaking, and do not diverge from each other up to 20 K, implying the $\omega\tau \ll 1$ regime is safely reached.

In the normal state, from the simple Drude model one can estimate the scattering lifetime τ from Z_s,

$$\frac{i\mu\omega}{Z_s^2} = \tilde{\sigma} = \frac{ne^2}{m^*}\frac{\tau}{1+i\omega\tau}. \tag{13}$$

Here, μ is the permeability, n is the electron density, m^* is the effective mass of the electron. One can regard the model as having two unknowns (ne^2/m^*, τ) and two equations (real and imaginary part), thus it is solvable at each temperature. The oscillator strength $ne^2/m^* = 2.33\times10^{18}$ $\mathrm{C^2m^{-3}kg^{-1}}$ is obtained. The obtained $\omega\tau(T)$ above T_c is shown in Supplementary Fig. 5. In the normal state above T_c, $\omega\tau \leq 0.05$ ($\tau \leq 0.88$ ps), satisfying the large scattering limit. Therefore, equating $R_s = X_s$ above 2 K ($\approx 1.25T_c$) in the case of the disk DR in Supplementary Note 2.D is further justified to a good approximation.

Note that the large scattering rate in the normal state $\Gamma_n(T > T_c) = 1/\tau(\sim 1$ THz$) \gg 2\Delta_0(0)(\sim 130$ GHz$)$ estimated above does not contradict the small bulk impurity scattering

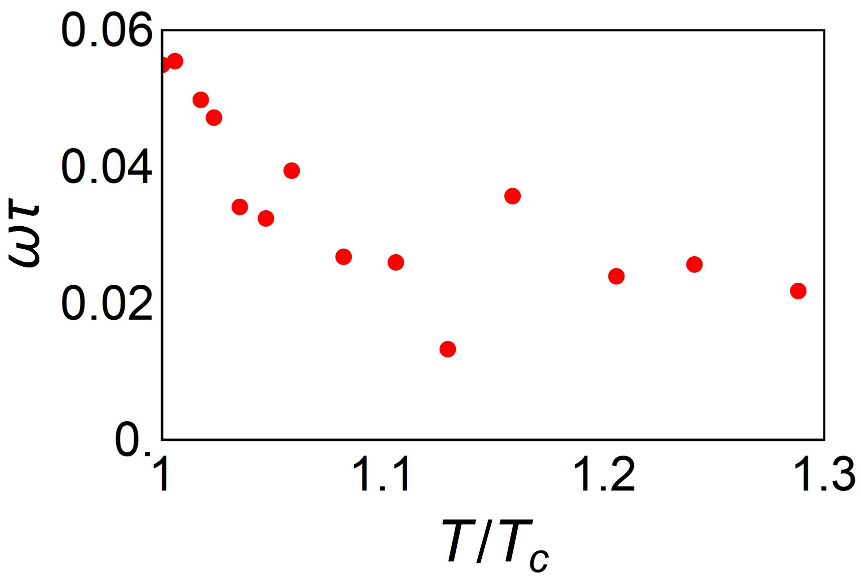

Supplementary Fig. 5. Extracted scattering time $\omega\tau$ from a Drude model analysis to the normal state complex conductivity data.

rate at low temperature $\Gamma_{\mathrm{imp}}(0) \leq 0.06\Delta_0(0)$ obtained from the superfluid density analysis (Discussion in the main text). The scattering rate in a superconductor consists of elastic scattering such as impurity scattering Γ_{imp}, and inelastic scattering such as electron-electron scattering Γ_{ee} (e.g. due to spin fluctuations), electron-phonon scattering $\Gamma_{\text{e-ph}}$ and other possible mechanisms. The experimentally obtained bulk scattering rate Γ_{tot} should be the sum of these various scattering rates $\Gamma_{\mathrm{tot}} = \Gamma_{\mathrm{imp}} + \Gamma_{\mathrm{ee}} + \Gamma_{\text{e-ph}} + \Gamma_{\mathrm{others}}$.

An important point here is that the scattering rates from different mechanisms can follow different temperature dependences across T_c. For example, while Γ_{imp} does not show noticeable difference across the T_c as seen from Ti (430/490 GHz for below/above T_c [21]), Γ_{ee} from spin fluctuations shows a very sharp drop just below T_c with a T^3 dependence. This sharp drop in Γ_{ee} was theoretically predicted [22] and experimentally observed in spin fluctuation dominating superconductors such as YBCO [23] and $CeCoIn_5$ [24]. As a result, for systems where the inelastic scattering mechanism dominates $\Gamma_{\mathrm{ee}} \gg \Gamma_{\mathrm{imp}}$ in the normal state, the ratio of the scattering rates in the normal and superconducting state ($\Gamma(T_c)/\Gamma(0)$) can reach up to 40 ($CeCoIn_5$) and 400 (YBCO). In this case, it is compatible to have a large total scattering rate in the normal state $\Gamma_{\mathrm{tot}}(T_c) = \Gamma_{\mathrm{imp}} + \Gamma_{\mathrm{ee}}(T_c) \approx \Gamma_{\mathrm{ee}}(T_c) \gg 2\Delta_0(0)$, and to have a low impurity scattering rate in the superconducting state $\Gamma_{\mathrm{tot}}^{\mathrm{bulk}}(0) \approx \Gamma_{\mathrm{imp}} \ll 2\Delta_0(0)$ at the same time.

UTe_2 is a system with the presence of strong magnetic fluctuations in the normal state as shown from the muon spin relaxation study and critical scaling behavior of the magnetism [14, 25]. Therefore, in UTe_2, it is possible that spin fluctuations dominate the scattering mechanism in the normal state $\Gamma_{\mathrm{ee}}(T_c) \gg \Gamma_{\mathrm{imp}}$. In this case, as shown in the above paragraph, having a large $\Gamma_{\mathrm{tot}}(T_c) \gg 2\Delta_0(0)$ in the normal state and being in the clean limit in the superconducting state $\Gamma_{\mathrm{imp}} \ll 2\Delta_0(0)$ can be reconciled due to the sharp drop in Γ_{ee} just below T_c.

SUPPLEMENTARY NOTE 4: ESTIMATION FOR THE MEAN FREE PATH AND JUSTIFICATION FOR LOCAL ELECTRODYNAMICS

When the measured surface impedance Z_s is converted to the complex conductivity, the simple relation $Z_s = \sqrt{i\mu_0\omega/\tilde{\sigma}}$ is used. This relation is valid in the local electrodynamics regime, which is $\xi(0) \ll \lambda(0)$ in the superconducting state and $l_{\mathrm{mfp}} \ll \delta_{\mathrm{skin}}$ in the normal state. Here, $\xi(0)$ is the superconducting coherence length at $T = 0$, $\lambda(0)$ is the magnetic penetration depth at $T = 0$, l_{mfp} is the mean free path, and δ_{skin} is the skin depth. To see if UTe_2 is indeed in this local regime, one needs to compare the values of l_{mfp}, δ_{skin}, $\xi(0)$, and $\lambda(0)$. First, for the superconducting state, $\xi(0)$ and $\lambda(0)$ are compared. Considering the estimated $\xi(0) = 4 \sim 7$ nm from H_{c2} measurement [14, 26] and $\lambda(0) \approx 791$ nm from the main text, we see that $\xi(0) \ll \lambda(0)$.

Second, for the normal state, since the scattering lifetime $\tau_n(T \geq T_c) \leq 0.88$ ps is obtained from Supplementary Fig. 5 (Supplementary Note 3), once the Fermi velocity v_F is known, $l_{\mathrm{mfp}} = v_F \tau_n$ can be easily estimated. In the recent angle-resolved photoemission spectroscopy (ARPES) study [27], the band Fermi velocity $v_F^{\mathrm{band}} \approx 3.9 \times 10^5$ m/s was obtained for the band along the k_x direction, which dominates the ARPES signal intensity. l_{mfp} estimated from this Fermi velocity is ≈ 340 nm. The normal state skin depth can be estimated from the electrical resistivity ρ_{tr} ($\delta_{\mathrm{skin}} = \sqrt{2\rho_{\mathrm{tr}}/\omega\mu}$). Using the ab-axis average of the ρ_{tr} reported in Ref. [14], δ_{skin} is estimated to be 2.74 μm at 1.8 K and 9.07 GHz, and hence $l_{\mathrm{mfp}} \ll \delta_{\mathrm{skin}}$. Therefore, UTe_2 is in the local electrodynamics regime both in the superconducting and normal state.

SUPPLEMENTARY NOTE 5: INCONSISTENCY BETWEEN SINGLET PAIRING STATES AND LOW TEMPERATURE BEHAVIOR OF THE PENETRATION DEPTH

As discussed in the Methods section, the penetration depth of UTe_2 shows a low temperature behavior which can be fit to $\Delta\lambda_{\rm eff}(T) = aT^c$ with $c = 2.11$. Here, the temperature exponent c is a good indicator for the nodal structure of the superconducting gap, which is closely related to the pairing state. In this section, the compatibility between the penetration depth data $\Delta\lambda_{\rm eff}(T)$ and various singlet pairing states will be examined.

The representative singlet pairing states are fully gapped s and line-nodal d-wave pairing states. First, the fully gapped s-wave state is expected to show an exponential temperature dependence with $c \geq 4$ power law at low temperature $T/T_c < 0.3$ [28] Second, a line-nodal $d_{x^2-y^2}$-wave pairing state is expected to show linear ($c \sim 1$) temperature dependence in the clean, local electrodynamics limit. Both of which are inconsistent with our data.

However, for the case of $d_{x^2-y^2}$-wave state, there are the possible cases which show a stronger than a linear temperature dependence (so that it is close to the observed quadratic behavior). The possible cases are a $d_{x^2-y^2}$ pairing state in the dirty limit [29], and $d_{x^2-y^2}$ state in the nonlocal electrodynamics regime [30]. In both cases, the low temperature behavior of the penetration depth shows a quadratic behavior below a crossover temperature T^*, and shows a linear behavior above T^*. This crossover behavior can be expressed as [29, 30],

$$\Delta\lambda_{\rm eff}(T) = \alpha\frac{T^2}{T+T^*}, \tag{14}$$

and hence the value of the temperature exponent for $\Delta\lambda_{\rm eff}(T)$ ranges from $1 \sim 2$ depending on the value of T^*.

The possibility of the dirty $d_{x^2-y^2}$-wave scenario can be examined by checking the value of T^*. To obtain a temperature dependence closed to quadratic, $T^*_{\rm imp}$ has to be much larger than T_c. For example, from Supplementary Equation 14, $T^*_{\rm imp} \geq 4T_c$ is required to obtain a power law exponent of $c \geq 1.95$. $T^*_{\rm imp}$ can be converted to an equivalent impurity scattering rate $\Gamma_{\rm imp} \simeq (k_B T^*_{\rm imp})^2/(0.83^2\Delta_0(0))$ [28]. If one assumes $\Delta_0(0) = 2.14k_BT_c$ which is a weakly-coupled BCS result of $d_{x^2-y^2}$ state, $T^*_{\rm imp} \geq 4T_c$ means $\Gamma_{\rm imp} \geq 10.86k_BT_c$. However a bulk impurity scattering rate much larger than the critical temperature will suppress the superconducting state even at zero temperature for a nodal superconductor [31, 32].

Therefore, the dirty limit $d_{x^2-y^2}$ pairing scenario is inconsistent with our data.

The possibility of a nonlocal electrodynamic regime can also be examined by the value of T^* which can be estimated from a superconducting coherence length $\xi(0)$, penetration depth $\lambda_{\text{eff}}(0)$, and the gap energy $\Delta(0)$ at zero temperature by $T^*_{\text{nonlocal}} = \Delta(0)\xi(0)/\lambda_{\text{eff}}(0)$ [30]. This estimation gives $T^*_{\text{nonlocal}} \approx 0.01T_c$ for our sample. This means that the penetration depth should follow the linear temperature dependence above $0.01T_c$, which is inconsistent with our data, which shows a quadratic behavior up to the full fitting range $0 \sim 0.3T_c$.

Another possibility for the d-wave pairing state other than $d_{x^2-y^2}$ is the $d_{zx}+id_{yz}$ pairing state. This pairing state possesses both point nodes and a line node. For example, one can imagine point nodes along the north and south pole, and a line node at the equatorial line of the superconducting Fermi surface. In this case, if the current in the sample is induced only along the point nodal direction, it may show the quadratic temperature dependence even in the presence of the line node. However, as discussed in Supplementary Note 2.D, the disk DR induces a circulating current in all directions in the ab-plane of the sample surface. Therefore, the response from the line nodal direction, which gives a linear $\Delta\lambda_{eff}(T)$, cannot be missed.

As just discussed. the above possibilities of representative singlet pairing states turn out to be inconsistent with our data. Also, the singlet pairing state cannot explain the reported upper critical field H_{c2} which is larger than the paramagnetic limiting field [14], and cannot explain the absence of a change in the NMR (nuclear magnetic resonance) Knight shift across T_c. [14, 33]. Therefore, in the superfluid density analysis of the main text, only spin-triplet pairing states are considered.

SUPPLEMENTARY NOTE 6: FURTHER QUALITATIVE EVIDENCE OF THE RESIDUAL NORMAL FLUID THROUGH THE TWO-FLUID MODEL

To further understand the anomalous residual σ_1, a two-fluid model for the electrodynamics can be introduced. For a homogenous superconducting medium, the two-fluid model describes the complex conductivity of the superconductor in terms of the superfluid and normal fluid contributions [18, 34],

$$\tilde{\sigma}(T) = \frac{ne^2}{m^*}\left(\frac{f_s(T)}{i\omega} + \frac{f_n(T)\tau_{\text{tot}}(T)}{1+i\omega\tau_{\text{tot}}(T)}\right), \tag{15}$$

where n is total electron density, m^* is the effective mass of the electrons, f_n is normal fluid fraction, $f_s = 1 - f_n$ is the superfluid fraction, and τ_{tot} is the effective normal fluid scattering lifetime. Note that $1/\tau_{\text{tot}}$ sums up scattering rates of various origins in a weighted manner, such as bulk impurity Γ_{imp}^{bulk} and topological surface normal fluid Γ^{surf} (if there is any).

From Supplementary Equation (15), the real and imaginary parts of the conductivity can be obtained as,

$$\sigma_1 = \frac{ne^2}{m^*}\frac{f_n \tau_{\text{tot}}}{1+\omega^2\tau_{\text{tot}}^2} \tag{16}$$

$$\sigma_2 = \frac{ne^2}{m^*}\frac{1+\omega^2\tau_{\text{tot}}^2 - f_n}{\omega(1+\omega^2\tau_{\text{tot}}^2)}. \tag{17}$$

If one divides Supplementary Equation (17) by (16), it yields a quadratic equation for $\omega\tau_{\text{tot}}$ in terms of the conductivity ratio σ_2/σ_1 and normal fluid fraction f_n with solution,

$$\omega\tau_{\text{tot}} = 0.5\left[\frac{\sigma_2}{\sigma_1}f_n \pm \sqrt{\left(\frac{\sigma_2}{\sigma_1}f_n\right)^2 - 4(1-f_n)}\right]. \tag{18}$$

To have real solutions for τ_{tot}, f_n must satisfy the following condition,

$$f_n \geq \frac{2}{1+\sqrt{1+(\sigma_2/\sigma_1)^2}} \tag{19}$$

If UTe_2 is a conventional superconductor without residual normal fluid, $\sigma_2/\sigma_1 \to \infty$ as $T \to 0$ [24, 35, 36], which yields $f_n \geq 0$, allowing the normal fluid to be fully depleted. However this is not the case for UTe_2. The complex conductivity data from the UTe_2 sample shows a finite value of $\sigma_2(0)/\sigma_1(0) = 2.22$. This value sets a non-vanishing lower limit for the residual normal fluid fraction $f_n(0) \geq 58$ %, not allowing full depletion of the normal fluid in the $T \to 0$ limit. Therefore, a conventional superconductor scenario cannot explain the $\tilde{\sigma}$ of UTe_2.

One important warning is that the above estimation of the residual normal fluid fraction should not be taken quantitatively, but instead, should be taken as qualitative evidence of the residual normal fluid. The reason is that the two-fluid model applies for a homogeneous medium, and requires modification for an inhomogeneous medium. For example, if one attributes the residual σ_1/σ_n to the surface normal fluid of a chiral superconductor and wants a more quantitatively accurate analysis, one needs to introduce a three-fluid model [19].

$$\tilde{\sigma}(\omega, T) = \frac{ne^2}{m^*}\left(\frac{f_s(T)}{i\omega} + \frac{f_{nb}(T)\tau_b(T)}{1+i\omega\tau_b(T)} + \frac{f_{ns}(T)\tau_s(T)}{1+i\omega\tau_s(T)}\right), \tag{20}$$

where b stands for bulk, and s stands for surface. However, this is an underdetermined system of equations (2 equations with 4 unknowns). Thus, one needs continuous frequency dependence data of the conductivity $\tilde{\sigma}(\omega)$, so that those unknowns can be estimated from fitting the frequency dependence. A microwave Corbino measurement on thin film samples [1, 2] is encouraged in the future for this purpose.

Also, the spatial profile of the microwave magnetic field and current inside the single crystal sample is complicated to quantitatively estimate. For example, if there are edges, the enhanced current distribution along the edges [37, 38] should be also considered to obtain a quantitatively rigorous estimation. For the case of a homogeneous superconductor, the spatial profile does not affect the procedure of estimating f_n and τ since these quantities are assumed to be uniform across the medium. However, for an inhomogeneous case such as the surface normal fluid and bulk superfluid, the complicated spatial profile of the field is a problem since it determines the weighting factor of the surface and bulk response. A simplified spatial profile, along with systematic control on it, and hence more quantitative study of the properties of the surface normal fluid, can be accomplished with development of superconducting thin films of UTe_2.

SUPPLEMENTARY NOTE 7: REMARKS ON THE RESIDUAL SPECIFIC HEAT RESULT

In the discussion of the main text, the absence of the linear term in the bulk thermal conductivity (κ/T) is cited as evidence of the absence of the bulk normal fluid [39]. However, it may be confusing to the readers because the initial specific heat study in Ref. [14] showed a residual linear term c/T which was, at that time, presented as a signature of bulk normal fluid. Here, we provide clarification on the result and interpretation of the c/T study.

The initial c/T data was taken above 400 mK. The later study [39] went down below 100 mK, and revealed that the high residual c/T value is due to the existence of a $1/T^{0.33}$ divergent term in c/T as $T \to 0$. The later study also revealed that the temperature dependence of the raw specific heat data (c/T vs T) does not obey the entropy balance condition which a superconductor must satisfy with this divergent term. Once the contribution from the divergent term in c/T is subtracted, the entropy balance is perfectly recovered. This observation implies that the divergent term in c/T as $T \to 0$ does not originate from electronic

	relative orientation	a	n	$\Delta_0(0)/k_B T_c$	σ_{RMS}
Axial state	$\mathbf{I} \parallel \mathbf{A}$	π^2	2	1.923	9.85×10^{-5}
	$\mathbf{I} \perp \mathbf{A}$	$7\pi^4/15$	4	0.969	1.72×10^{-2}
Polar state	$\mathbf{I} \parallel \mathbf{A}$	$27\pi\zeta(3)/4$	3	1.284	5.82×10^{-3}
	$\mathbf{I} \perp \mathbf{A}$	$3\pi \ln 2/2$	1	6.285	1.41×10^{-2}

Supplementary Table 1. (First four columns) Low temperature theoretical asymptotes of the normalized superfluid density $\rho_s(T) = 1 - a(k_B T/\Delta_0)^n$ with the predicted values for parameters (a, n) for the various spin-triplet pairing states, and the relative direction between the symmetry axis of the gap $\mathbf{I}$ and the direction of the vector potential $\mathbf{A}$ (which is same as that of the current) [28, 40]. (Last two columns) The results of fitting the $\rho_s(T)$ data with the theoretical asymptotes of each scenario. Here, $\Delta_0(0)/k_B T_c$ was the single fitting parameter and σ_{RMS} is the root-mean-square error of the fit.

entropy. Therefore, the residual c/T due to the divergent term should not be attributed to bulk normal electronic states. Examination of other possible origins such as magnetic entropy or quantum critical fluctuations might be an interesting future research direction.

SUPPLEMENTARY NOTE 8: DETAILS OF THE FITTING PARAMETER IN THE NORMALIZED SUPERFLUID DENSITY ANALYSIS

In this section, details of the normalized superfluid density $\rho_s(T)$ fit for the spin-triplet pairing states will be introduced for the reader's convenience. Note that one can also refer to Ref. [28, 40] for broader context and details. In general, if the symmetry axis of the gap is denoted as $\hat{\mathbf{I}}$, $\rho_s(T)$ for the case of $\hat{\mathbf{I}} \parallel \hat{\mathbf{A}}$ and $\hat{\mathbf{I}} \perp \hat{\mathbf{A}}$ can be calculated as,

$$\rho_s(T)_{(\parallel,\perp)} = 1 - 3n \int_0^1 d\cos\theta (\cos^2\theta, \frac{1}{2}\sin^2\theta) \int_{-\infty}^{+\infty} d\epsilon_k \left(-\frac{\partial f(E_k)}{\partial E_k} \right), \tag{21}$$

where $E_k = \sqrt{\epsilon_k^2 + \Delta_k^2}$. In the case of the spin-triplet states, at low temperature the $\rho_s(T)$ has simpler theoretical asymptotes $\rho_s(T) = 1 - a(k_B T/\Delta_0)^n$. The values of the fitting parameter $\Delta_0(0)/k_B T_c$ from fitting the $\rho_s(T)$ data into these theoretical forms are shown in the main text Fig. 3(c) and Supplementary Table 1).

Here, the $\rho_s(T)$ data for $T \leq 0.26T_c$ was fitted. The reason this temperature range is chosen is that the maximum gap magnitude $\Delta_0(T)$ is nearly constant for $T \leq 0.26T_c$ [28].

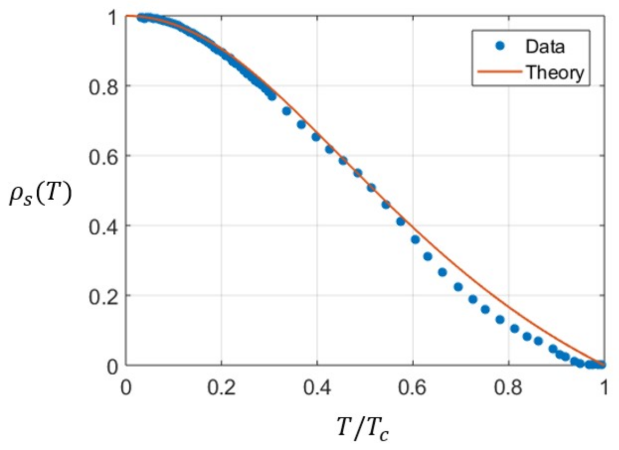

Supplementary Fig. 6. The $\rho_s(T)$ data of the UTe_2 sample for the full temperature range (0 to T_c) and a comparison to the theoretical estimation (Supplementary Equation (21)), using experimentally determined $\Delta_0(0)/k_B T_c$ and $\Delta c/c$ as discussed in the text.

One can estimate the temperature dependence of the gap by [40],

$$\Delta_0(T) = \Delta_0(0) \tanh \left(\frac{\pi k_B T_c}{\Delta_0(0)} \sqrt{\frac{2}{3} \frac{\Delta c}{c} \left(\frac{T_c}{T} - 1 \right)} \right), \tag{22}$$

where $\Delta c/c$ is the normalized jump in specific heat at T_c. $\Delta_0(T)$ shows only a 1.8 % decrease between 0 to 0.26 T_c. Therefore, for $T \leq 0.26 T_c$, $\Delta_0(T) \approx \Delta_0(0)$ so that one does not need to include $\Delta c/c$ as a fit parameter. The fewer the number of fitting parameters, the less arbitrary (and more accurate) the estimated parameter values are. This is the main reason why the superfluid density studies have been conducted effectively at $T \leq 0.26 T_c$ regime for the pairing state analysis [28].

To satisfy the reader's curiosity, if one plots the $\rho_s(T)$ data over the full temperature range (the same data displayed in Fig. 3(c) of the main text) with the theoretical estimation from Supplementary Equation (21) using $\Delta c/c = 1.18$ (obtained after subtracting the non-electronic contribution (from the data in Ref. [39])) and $\Delta_0(0)/k_B T_c = 1.923$ (obtained from the low temperature range $\rho_s(T)$ study), the data and the theory show good agreement (see Supplementary Fig. 6).

SUPPLEMENTARY NOTE 9: INSULATING PROPERTIES OF URANIUM OXIDES

The UTe_2 sample has been prepared and handled in a nitrogen environment, such as a glove box, to exclude the potential effect of oxidizing the sample surface. Nevertheless, a thorough survey of the electrical properties of uranium oxides was conducted to address any concern that a metallic phase could exist on the surface. The early study of the electrical properties of the oxidized uranium was done on UO_2, which shows it is a semiconductor with a large band gap ~ 2.1 eV [41]. Another comprehensive study of the chemical constituents and their electronic properties of uranium oxide films shows that all of the common oxides of uranium (UO_2, U_4O_9, U_3O_7, U_3O_8, α-UO_3, δ-UO_3, γ-UO_3) are semiconductors with band gaps in the 1 to 3 eV range [42]. Similar results were recently obtained on epitaxial UO_2, α-U_3O_8, and α-UO_3 thin films [43]. At the temperatures of concern here (< 3 K, $k_BT < 0.26$ meV), they should all show an insulating response. This makes the oxidized surface, if there is any, less likely to produce the observed microwave dissipation and corresponding residual normal metallic behavior in the $T \to 0$ limit at 11 GHz ($\hbar\omega = 0.044$ meV).

SUPPLEMENTARY NOTE 10: EFFECT OF N-GREASE ON THE SURFACE IMPEDANCE STUDY OF THE SAMPLE

In our study, we applied Apeizon N-grease between the sample and its thermal reservoir. The N-grease is the product name of a purified hydrocarbon low temperature adhesive that ensures thermal connection between the sample and the thermal reservoir at cryogenic temperatures, while being electrically insulating (https://apiezon.com/products/vacuum-greases/apiezon-n-grease/). Hence, it has been widely used for various microwave experiments in cryogenic conditions. To make sure that it didn't affect our microwave impedance measurement of the sample, we compared the $1/Q$ with and without a drop of N-grease present (which is the same amount we apply when we mount the sample) with the disk dielectric resonator setup. We only see a change in $1/Q$ of $\approx 10^{-6}$, which is near the resolution limit of our 1/Q measurement. This is marginal compared to 1/Q in the presence of the sample ($10^{-3} \sim 10^{-4}$). Therefore, we concluded the N-grease does not affect our results with the sample.

SUPPLEMENTARY NOTE 11: EXAMINATION OF TOPOLOGICALLY TRIVIAL ORIGINS OF THE SURFACE NORMAL FLUID

From the theoretical point of view, it is proposed that a rough surface can suppress the order parameter of an anisotropic superconductor through diffusive scattering at the disordered surface [44] with thickness d smaller than coherence length $\xi(0)$ [45]. The surface disorder provides a shorter mean free path l_{mfp} than the bulk, resulting in a large, dominant surface impurity scattering rate $\Gamma_{\mathrm{imp}}^{\mathrm{surf}}$. Once $\Gamma_{\mathrm{imp}}^{\mathrm{surf}}$ exceeds a certain critical value, the order parameter at such a surface can be suppressed. However, Γ_{imp} does not show noticeable temperature dependence as seen from Ti (430/490 GHz for below/above T_c [21]). This implies that if a surface normal fluid is introduced by a dominant $\Gamma_{\mathrm{imp}}^{\mathrm{surf}}$ from the disorder of the rough surface, its contribution to σ_1 would not provide the more than factor of 2 enhancement ($\sigma_1(0)/\sigma_n > 2$) observed in our data.

Another topologically trivial origin for the surface normal fluid is the case of the clean surface with specular reflection. The specular reflection can break Cooper pairs in a superconductor whose order parameter changes sign for different directions of the momentum before and after the reflection. Theoretical studies expect this effect for d-wave superconductors to create a normal fluid region (Andreev bound state - ABS) at its surface with a thickness $L \sim \xi(0)$ [46]. The consequent experimental signatures of this surface ABS were observed as a zero-bias conductance peak in YBCO by point-contact spectroscopy [47].

However, the microwave conductivity studies of such systems [23, 48] do not show the monotonic increase in σ_1 below T_c observed in our study (Fig. 2(b) in main text). Instead, they show a significant decrease in σ_1 as $T \to 0$ as seen from Fig. 9 of Ref. [48]). This is possibly because the thickness of the normal layer from ABS is very thin ($\xi(0) \approx 2$ nm [49]) compared to penetration depth $\lambda(0) \approx 155$ nm [50] which determines the probing depth of the electromagnetic response. In this case, the normal layer contribution to σ_1 can be marginal. Similarly, UTe_2 has $\xi(0) = \sqrt{\Phi_0/2\pi H_{c2}} = 3 \sim 7$ nm [14] and $\lambda(0) \approx 791$ nm, showing even a smaller ratio of $\xi(0)/\lambda(0)$ than YBCO. This fact makes the contribution of the normal fluid layer from the ABS to σ_1, even if the layer exists, really marginal. Note that the same thickness argument applies for the effect of a surface disordered layer on σ_1.

On the other hand, a naïve, but effective estimation of the thickness of the chiral surface state (d_{css}) can be given by the uncertainty principle $\Delta E \Delta t \sim \hbar/2$. As $\Delta t = d_{css}/v_F$ and

$\Delta E \sim \Delta_0(0)$, one obtains $d_{css} = \hbar v_F/2\Delta_0(0)$. With $v_F \approx 3.9 \times 10^5$ m/s from ARPES study [27] and $\Delta_0 \approx 0.265$ meV from superfluid conductivity analysis in this work (and STM spectra [51]), d_{css} is estimated to be 488 nm, which is comparable to $\lambda(0) \approx 791$ nm. The chiral surface normal state with the thickness d_{css} comparable to $\lambda(0)$ could certainly provide significant contribution to the normal fluid response $\sigma_1(T)$. Note that the large residual normal fluid fraction $f_n(0) \approx 0.58$ calculated from the two-fluid model in Supplementary Note 6 (Although it is only a qualitative analysis) is similar to the ratio $d_{css}/\lambda(0) = 0.62$, providing more support to the scenario of the large normal fluid response from the chiral surface state. A qualitative argument explaining the monotonic increase of $\sigma_1(T)$ as $T \to 0$ is provided in the paragraph just before the conclusion of the main text.

Supplementary References

[1] Booth, J. C., Wu, D. H. & Anlage, S. M. A broadband method for the measurement of the surface impedance of thin films at microwave frequencies. *Rev. Sci. Instrum.* **65**, 2082–2090 (1994). URL `https://doi.org/10.1063/1.1144816`.

[2] Scheffler, M., Dressel, M., Jourdan, M. & Adrian, H. Extremely slow Drude relaxation of correlated electrons. *Nature* **438**, 1135–1137 (2005). URL `https://doi.org/10.1038/nature04232`.

[3] Petersan, P. J. & Anlage, S. M. Measurement of resonant frequency and quality factor of microwave resonators: Comparison of methods. *J. Appl. Phys.* **84**, 3392–3402 (1998). URL `https://doi.org/10.1063/1.368498`.

[4] Bae, S., Lee, S., Zhang, X., Takeuchi, I. & Anlage, S. M. Microwave meissner screening properties of proximity-coupled topological-insulator / superconductor bilayers. *Phys. Rev. Materials* **3**, 124803 (2019). URL `https://link.aps.org/doi/10.1103/PhysRevMaterials.3.124803`.

[5] Kajfez, D. *Q factor* (Vector Fields, 1994).

[6] Matthaei, G. L. & Hey-Shipton, G. L. Concerning the use of high-temperature superconductivity in planar microwave filters. *IEEE Trans. Microw. Theory Tech* **42**, 1287–1294 (1994).

[7] Ghigo, G. *et al.* Effects of heavy-ion irradiation on the microwave surface impedance of $(Ba_{1-x}K_x)Fe_2As_2$ single crystals. *Supercond. Sci. Technol.* **31**, 34006 (2018). URL `http://dx.doi.org/10.1088/1361-6668/aaa858`.

[8] Huttema, W. A. *et al.* Apparatus for high-resolution microwave spectroscopy in strong magnetic fields. *Rev. Sci. Instrum.* **77**, 023901 (2006). URL `https://doi.org/10.1063/1.2167127`.

[9] Hein, M. A. *High-Temperature Superconductor Thin Films at Microwave Frequencies* (Springer, Heidelberg, 1999).

[10] Bonn, D. A. & Hardy, W. N. *Microwave Electrodynamics of High Temperature Superconductors* (Springer New York, New York, NY, 2007). URL `https://doi.org/10.1007/978-0-387-68734-6_4`.

[11] Zhang, X. *Microwave Flux-Flow Impedance Measurement of Type-II Superconductors* (Simon

Fraser University, 2008). URL https://summit.sfu.ca/item/9887.

[12] Tobar, M. E., Krupka, J., Ivanov, E. N. & Woode, R. A. Anisotropic complex permittivity measurements of mono-crystalline rutile between 10 and 300 K. *Journal of Applied Physics* **83**, 1604–1609 (1998). URL https://doi.org/10.1063/1.366871.

[13] Bae, S. *et al.* Dielectric resonator method for determining gap symmetry of superconductors through anisotropic nonlinear meissner effect. *Rev. Sci. Instrum.* **90**, 043901 (2019). URL https://doi.org/10.1063/1.5090130.

[14] Ran, S. *et al.* Nearly ferromagnetic spin-triplet superconductivity. *Science* **365**, 684–687 (2019). URL https://science.sciencemag.org/content/365/6454/684.

[15] Sridhar, S. & Kennedy, W. L. Novel technique to measure the microwave response of high T_c superconductors between 4.2 and 200 K. *Review of Scientific Instruments* **59**, 531–536 (1988). URL https://doi.org/10.1063/1.1139881.

[16] Rubin, D. L. *et al.* Observation of a narrow superconducting transition at 6 GHz in crystals of $YBa_2Cu_3O_7$. *Phys. Rev. B* **38**, 6538–6542 (1988). URL https://link.aps.org/doi/10.1103/PhysRevB.38.6538.

[17] Murphy, N. *Microwave frequency vortex dynamics of the heavy fermion superconductor $CeCoIn_5$*. Master's thesis (2010). URL http://summit.sfu.ca/item/12487.

[18] Ormeno, R. J., Sibley, A., Gough, C. E., Sebastian, S. & Fisher, I. R. Microwave Conductivity and Penetration Depth in the Heavy Fermion Superconductor $CeCoIn_5$. *Phys. Rev. Lett.* **88**, 047005 (2002). URL https://link.aps.org/doi/10.1103/PhysRevLett.88.047005.

[19] Ormeno, R. J. *et al.* Electrodynamic response of Sr_2RuO_4. *Phys. Rev. B* **74**, 092504 (2006). URL https://link.aps.org/doi/10.1103/PhysRevB.74.092504.

[20] Nelson, W. E. & Hoffman, A. R. *Measurements of the Temperatures and Magnetic Field Dependence of Electrical Resistivity and Thermal Conductivity in OFHC Copper*, 73–80 (Springer US, Boston, MA, 1976). URL https://doi.org/10.1007/978-1-4899-3751-3_13.

[21] Thiemann, M., Dressel, M. & Scheffler, M. Complete electrodynamics of a BCS superconductor with μeV energy scales: Microwave spectroscopy on titanium at mK temperatures. *Phys. Rev. B* **97**, 214516 (2018). URL https://link.aps.org/doi/10.1103/PhysRevB.97.214516.

[22] Quinlan, S. M., Scalapino, D. J. & Bulut, N. Superconducting quasiparticle lifetimes due to spin-fluctuation scattering. *Phys. Rev. B* **49**, 1470–1473 (1994). URL https://link.aps.

org/doi/10.1103/PhysRevB.49.1470.

[23] Bonn, D. A. *et al.* Microwave determination of the quasiparticle scattering time in $YBa_2Cu_3O_{6.95}$. *Phys. Rev. B* **47**, 11314–11328 (1993). URL https://link.aps.org/doi/10.1103/PhysRevB.47.11314.

[24] Truncik, C. J. S. *et al.* Nodal quasiparticle dynamics in the heavy fermion superconductor CeCoIn5 revealed by precision microwave spectroscopy. *Nat. Commun.* **4**, 2477 (2013). URL https://doi.org/10.1038/ncomms3477.

[25] Sundar, S. *et al.* Coexistence of ferromagnetic fluctuations and superconductivity in the actinide superconductor UTe_2. *Phys. Rev. B* **100**, 140502 (2019). URL https://link.aps.org/doi/10.1103/PhysRevB.100.140502.

[26] Aoki, D. *et al.* Unconventional Superconductivity in Heavy Fermion UTe2. *J. Phys. Soc. Jpn.* **88**, 043702 (2019). URL https://doi.org/10.7566/JPSJ.88.043702.

[27] Miao, L. *et al.* Low Energy Band Structure and Symmetries of UTe_2 from Angle-Resolved Photoemission Spectroscopy. *Phys. Rev. Lett.* **124**, 076401 (2020). URL https://link.aps.org/doi/10.1103/PhysRevLett.124.076401.

[28] Prozorov, R. & Giannetta, R. W. Magnetic penetration depth in unconventional superconductors. *Supercond. Sci. Technol.* **19**, R41 (2006). URL http://stacks.iop.org/0953-2048/19/i=8/a=R01.

[29] Hirschfeld, P. J. & Goldenfeld, N. Effect of strong scattering on the low-temperature penetration depth of a d-wave superconductor. *Phys. Rev. B* **48**, 4219–4222 (1993). URL https://link.aps.org/doi/10.1103/PhysRevB.48.4219.

[30] Chia, E. E. M. *et al.* Nonlocality and strong coupling in the heavy fermion superconductor $CeCoIn_5$: A penetration depth study. *Phys. Rev. B* **67**, 014527 (2003). URL https://link.aps.org/doi/10.1103/PhysRevB.67.014527.

[31] Radtke, R. J., Levin, K., Schüttler, H.-B. & Norman, M. R. Predictions for impurity-induced T_c suppression in the high-temperature superconductors. *Phys. Rev. B* **48**, 653–656 (1993). URL https://link.aps.org/doi/10.1103/PhysRevB.48.653.

[32] Mackenzie, A. P. *et al.* Extremely Strong Dependence of Superconductivity on Disorder in Sr_2RuO_4. *Phys. Rev. Lett.* **80**, 161–164 (1998). URL https://link.aps.org/doi/10.1103/PhysRevLett.80.161.

[33] Nakamine, G. *et al.* Superconducting Properties of Heavy Fermion UTe2 Revealed by 125Te-

nuclear Magnetic Resonance. *J. Phys. Soc. Jpn* **88**, 113703 (2019). URL https://doi.org/10.7566/JPSJ.88.113703.

[34] Tinkham, M. *Introduction to Superconductivity, 2nd edition* (Dover publications, Inc., 1996).

[35] Glover, R. E. & Tinkham, M. Conductivity of superconducting films for photon energies between 0.3 and $40kT_c$. *Phys. Rev.* **108**, 243–256 (1957). URL https://link.aps.org/doi/10.1103/PhysRev.108.243.

[36] Klein, O., Nicol, E. J., Holczer, K. & Grüner, G. Conductivity coherence factors in the conventional superconductors Nb and Pb. *Phys. Rev. B* **50**, 6307–6316 (1994). URL https://link.aps.org/doi/10.1103/PhysRevB.50.6307.

[37] Culbertson, J. C., Newman, H. S. & Wilker, C. Optical probe of microwave current distributions in high temperature superconducting transmission lines. *J. Appl. Phys.* **84**, 2768–2787 (1998). URL http://dx.doi.org/10.1063/1.368390.

[38] Zhuravel, A. P., Ustinov, A. V., Harshavardhan, K. S. & Anlage, S. M. Influence of LaAlO3 surface topography on rf current distribution in superconducting microwave devices. *Applied Physics Letters* **81**, 4979–4981 (2002). URL http://dx.doi.org/10.1063/1.1530753.

[39] Metz, T. *et al.* Point-node gap structure of the spin-triplet superconductor UTe_2. *Phys. Rev. B* **100**, 220504 (2019). URL https://link.aps.org/doi/10.1103/PhysRevB.100.220504.

[40] Gross, F. *et al.* Anomalous temperature dependence of the magnetic field penetration depth in superconducting UBe_{13}. *Z. Phy. B* **64**, 175–188 (1986). URL https://doi.org/10.1007/BF01303700.

[41] Schoenes, J. Optical properties and electronic structure of UO_2. *J. Appl. Phys.* **49**, 1463–1465 (1978). URL https://doi.org/10.1063/1.324978.

[42] He, H., Andersson, D. A., Allred, D. D. & Rector, K. D. Determination of the insulation gap of uranium oxides by spectroscopic ellipsometry and density functional theory. *J. Phys. Chem. C* **117**, 16540–16551 (2013). URL https://doi.org/10.1021/jp401149m.

[43] Enriquez, E. *et al.* Structural and Optical Properties of Phase-Pure UO_2, α-U_3O_8, and α-UO_3 Epitaxial Thin Films Grown by Pulsed Laser Deposition. *ACS Appl. Mater. Interfaces* **12**, 35232–35241 (2020). URL https://doi.org/10.1021/acsami.0c08635.

[44] Barash, Y. S., Svidzinsky, A. A. & Burkhardt, H. Quasiparticle bound states and low-temperature peaks of the conductance of nis junctionsin d-wave superconductors. *Phys. Rev. B* **55**, 15282–15294 (1997). URL https://link.aps.org/doi/10.1103/PhysRevB.55.15282.

[45] Bakurskiy, S. V. *et al.* Local impedance on a rough surface of a chiral p-wave superconductor. *Phys. Rev. B* **98**, 134508 (2018). URL https://link.aps.org/doi/10.1103/PhysRevB.98.134508.

[46] Löfwander, T., Shumeiko, V. S. & Wendin, G. Andreev bound states in high-Tc superconducting junctions. *Supercond. Sci. Technol* **14**, R53–R77 (2001). URL https://doi.org/10.1088%2F0953-2048%2F14%2F5%2F201.

[47] Aprili, M., Badica, E. & Greene, L. H. Doppler Shift of the Andreev Bound States at the YBCO Surface. *Phys. Rev. Lett.* **83**, 4630–4633 (1999). URL https://link.aps.org/doi/10.1103/PhysRevLett.83.4630.

[48] Hosseini, A. *et al.* Microwave spectroscopy of thermally excited quasiparticles in $YBa_2Cu_3O_{6.99}$. *Phys. Rev. B* **60**, 1349–1359 (1999). URL https://link.aps.org/doi/10.1103/PhysRevB.60.1349.

[49] Varshney, D., Singh, R. K. & Shah, S. Coherence lengths and magnetic penetration depths in $YBa_2Cu_3O_7$ and $YBa_2Cu_4O_8$ superconductors. *J. Supercon.* **9** (1996-12).

[50] Tallon, J. L. *et al.* In-Plane Anisotropy of the Penetration Depth Due to Superconductivity on the Cu-O Chains in $YBa_2Cu_3O_{7-\delta}$, $Y_2Ba_4Cu_7O_{15-\delta}$, and $YBa_2Cu_4O_8$. *Phys. Rev. Lett.* **74**, 1008–1011 (1995). URL https://link.aps.org/doi/10.1103/PhysRevLett.74.1008.

[51] Jiao, L. *et al.* Chiral superconductivity in heavy-fermion metal UTe2. *Nature* **579**, 523–527 (2020). URL https://doi.org/10.1038/s41586-020-2122-2.
